# Water-Handling Patterns and Associated Microbial Profiles in relation to Hygiene in Babati Town, Tanzania

**DOI:** 10.1155/2019/5298247

**Published:** 2019-05-20

**Authors:** Irene Tesha, Revocatus Machunda, Karoli Njau, Emmanuel Mpolya

**Affiliations:** ^1^School of Life Sciences and Bioengineering (LiSBE), Nelson Mandela African Institution of Science and Technology (NM-AIST), P.O. Box 447, Arusha, Tanzania; ^2^School of Materials, Energy, Water and Environmental Sciences (MEWES), Nelson Mandela African Institution of Science and Technology (NM-AIST), P.O. Box 447, Arusha, Tanzania

## Abstract

**Introduction:**

In rapidly urbanizing centres in Tanzania, water supply infrastructure lags behind the speed of urbanization, affecting water availability and accessibility. We believe that inhabitants' access water using various ways which are characterizable and understanding them could inform about the risks to hygiene-related diseases. This study aimed at characterizing water-handling chains and their microbial profiles in Babati town to inform hygiene education policy and water supply planning.

**Methodology:**

A cross-sectional study design employing a proportional sampling for each of the 8 wards was conducted between November 2016 and March 2017. A total of 564 samples of water were collected using the USA EPA procedures from 37 randomly selected households. Water samples were collected from the common sources of water as well as from the downstream points to multiple storage containers. Using EPA membrane filtration techniques, two microorganisms were tested: fecal coliforms and *Salmonella typhi. Results*. Three water-handling chains/patterns in Babati town were determined, and they were as follows: (i) untreated-source-to-treated-reservoir-to-households (*untrS2trR2HH*) chain, (ii) untreated-source-to-untreated-reservoir-to-households (*untrS2untrR2HH*) chain; (iii) untreated-source-straight-to-households (*untrS2HH*) chain. In terms of the microbial profile, the most contaminated water-handling chain was the untreated-source-straight-to-households (*untrS2HH*). The number of users in these three chains was not statistically significantly different (*p*=0.5226), meaning that all people utilized the various chains almost equally, depending on the water situation. Most households (83%) did not treat their drinking water making those using the untreated-source-to-household chain (*untrS2HH*) most vulnerable to waterborne diseases.

**Conclusion:**

Determination of water-handling chains among the household is a novel approach which allows an understanding of the points at which highest fecal loading occurs. This approach therefore may inform the development of policies in the areas of household hygiene education, drinking water treatment, and water supply planning in urbanized towns in Tanzania and other developing countries.

## 1. Background

Water-handling practices are important determinants of the role of water in disease transmission. Water handling refers to various steps taken by households in securing water from source to consumption which include how water is collected, transported, stored, and eventually used [1]. While water is one of the precious gifts to mankind, lack of access to safe drinking water and basic sanitation is one of the problems affecting billions of people around the world [2]. This is particularly so in developing countries where levels of access to water and water-related facilities are low as more than one billion people in these countries lack access to safe water [3, 4].

Poor access to water is at the heart of the poverty trap especially for women and children who suffer in terms of illness and lost opportunities as a result of spending long times searching for water [5]. In Tanzania, according to the demographic and health survey of 2015/2016, 6 in 10 (61%) households have access to an improved water source (DHS et al., 2015/2016). Among urban mainland households, 86% have access to an improved water source compared to 48% of rural mainland households. Almost 2 in 10 households (19%) in Tanzania have an improved, nonshared sanitation facility. In rural areas on the mainland, the majority (86%) of households have unimproved sanitation facilities, while in urban mainland areas, only 23% of households have unimproved facilities [6].

One of the major causes of waterborne diseases that contribute both morbidity and mortality in children and adults in Tanzania is water pollution [7]. Diarrheal diseases with 81.0 million [70.1–97.2] DALYs have been one of the hierarchy greatest contributors to global DALYs in 2017 among communicable, maternal, neonatal, and nutritional disorders at Level 3 of the global burden diseases [8]. In Tanzania, waterborne diseases account for 23,900 deaths of children under five years of age per year [9]. According to the 2010 Tanzania Demographic and Health Survey, about 60% of households in Tanzania do not treat their water; therefore, improving quality at the source of water alone does not always decrease risks of diseases because subsequent contamination after collection and during storage reverses the advantages of water improvement at the source only [5]. In general, the contamination levels are substantially higher in household water containers than in water taps [10]. Children may in particular cause contamination when they put their fecally contaminated hands or utensils into the household water containers [11].

Understanding water-handling chains and their microbial profiles is important towards elimination of waterborne diseases. Most waterborne diseases cause diarrheal illnesses that are attributed to unsafe water supply as well as inadequate hygiene and sanitation. In developing countries, the most common problem affecting under-five children is diarrhea [12]. Majority of diarrhea cases worldwide (up to 80%) are linked to unsafe water, inadequate sanitation, or insufficient hygiene [13]. Poor sanitation and hygiene account for 7% of deaths in low- and middle-income countries [14]. Hygiene-related diseases, like diarrhea as the second leading cause of death in low-income countries in the year 2004, kill around 1.5 million people every year with infant mortality being high in developing countries [15] where around 80% of people worldwide who die from diarrheal disease are children below five years of age [16]. Hygiene interventions that target the adult females at the household may help develop household hygienic practices and transform habits of children [17]. Women are responsible for collecting, storing, and treating water in many homes [18] and also have a significant role to play for the children's safety. In areas with scarcity of water, these roles of women become even more important to the overall survival of children [19]. A high proportion of children have been observed to collect and serve water, but it is presumed that they are less careful in avoiding hand contact [17]. Hand washing as a hygienic behavior is one of the most important factors in stopping the spread of microbial contamination and staying healthy [20]. Unwashed hands can accelerate the spread of bacteria, parasites, and viruses that are transmitted from human and animal faeces or the environment [1]. Washing hands after using the bathroom, before and after preparing and eating food, whenever hands are visibly soiled, and more frequently during times of illness can help stop the spread of disease from one person to another [21].

The presence of bacteria such as *Escherichia coli* and *Salmonella* in water is one of the root causes of various diseases and infections. Their presence indicates that the water may be contaminated with human or animal wastes [22]. These bacteria can be found in various water sources which have contact with animals or humans, such as rivers, tanks, taps, and wells and are known to be able to survive for up to more than two weeks [23]. Contamination in these waters can cause illnesses such as diarrhea, cramps, nausea, and headaches, to mention a few. In particular, these contaminations are more dangerous to infants, young children, the elderly, and people who are severely immune compromised [22]. In fact, it has been reported elsewhere that the illness and mortality due to waterborne salmonellosis is on the increase in developing countries [23].

Microbial contamination of collected and stored household water is caused not only by the collection and use of fecally contaminated water that was not safe to begin with but also by contamination of microbiologically safe water after its collection and storage [10]. This study focused on investigating water handling and storage practices among inhabitants of the Babati town in Tanzania in order to characterize those water-handling patterns or chains and investigate the various hygienic practices in each chain as well as their microbial profiles so as to understand the possible health risks with each pattern or chain [4]. Tracking microbial drinking water quality along different water supply “chains” to arrival in the household is a novel approach which allows for an understanding of the points at which highest fecal loading occurs. This approach thereby assists to inform the development of policies in the areas of household hygiene education, drinking water treatment, and water supply planning in rapidly growing urbanized towns in Tanzania and elsewhere in developing countries.

## 2. Methods

### 2.1. Study Location

This study was conducted within the 8 wards which constitute the Babati town council, namely, Bagara, Maisaka, Bonga, Mutuka, Singe, Sigino, Nangara, and Babati [24]. Babati town lies between latitudes 3°S and 4°S and between longitudes 35°E and 36°E [25]. According to the National Census of 2012, the town covers an area of 471.33 square kilometers and density of 197.5 inhabitants per square kilometers and had a population status of 93,108 people. Out of which 47,313 were male, and 45,795 were females [24]. The main primary economic activities in Babati town are fishing, livestock keeping, tourism, and agriculture production. Lying along the shores of the Lake Babati and being surrounded by small mountains the area is potentially vulnerable to environmental and water pollutions due to an increase in urbanization and social economic activities around the lake.

### 2.2. Study Design, Sampling, and Sample Size

In this study, the unit of analysis was a water sample collected from various water collection points along the source-to-consumption water-handling chain. We employed a cross-sectional study which was conducted from November 2016 to March 2017 using a proportional sampling as follows: Babati town has eight wards listed from the largest to the smallest: Babati, Bagara, Maisaka, Nangara, Sigino, Bonga, Singe, and Mutuka. In this proportional sampling design, a random sample of households per ward was selected by applying the weighting factor depending on the total population size of the particular ward as follows: seven households from the Babati ward, six households from Bagara, and then four households from each of Maisaka, Nangara, Singe, Bonga, Sigino, and Mutuka. This made a random sample of 37 households. At the last level of sampling, water-handling points among these 37 households had their water sampled to produce our unit of analysis. The number of water collection points which would be powerful to detect any differences in the water-handling chains if they existed was calculated as follows [26].

(1) $$\begin{array}{c} n=\frac{\text{deff}}{r}\times \frac{z^{2}P\left(1-P\right)}{e^{2}}. \end{array}$$

Since the population of Babati at the time was about 93,000, the population correction factor was dropped as it evaluated to 1. In this formula, *d*_*eff*_ is the design effect which is equal to 1.5, *r* is the response rate which is equal to 1, *z* is the 95% percentile point of the normal distribution in which 95% of the area of the curve lies which is equal to 1.96, and *p*=0.5, which is the value of the binomial probability at maximum variance (for maximum sample size). Finally, *e* is the precision, which was set at 5%. This yielded a sample size of 564.

### 2.3. Data Collection

All data were collected by the first author under the supervision of all coauthors. The first author is professionally a Laboratory Scientist at NM-AIST. According to the EPA standard method of water sample collection [27], water samples were collected from the various points constituting different patterns, which included water sources, reservoirs, and taps and household's multiple storage containers for bacterial analysis. All water samples were collected in triplicate to test for two organisms (fecal coliforms and *Salmonella typhi*). Colony counts were analyzed and reported as CFU/100 mls and plotted using a logarithmic scale. On sampling from open ground water sources, the inverted containers were immersed beneath the water surface and turned upright before removal to minimize surface contamination. Tap water sources were sampled after allowing the water to run for 20–30 seconds. Samples were collected using ThermoFisher Scientific™ sterile containers (which are sterile until opened for sampling according to the manufacturer) [28]. Containers were labeled and transported in cooler bags to the laboratory. Sample processing started within six to eight hours after collection. If incubation was delayed beyond 48 hours, the sample was discarded because multiplication or death or competing organisms might interfere with coliform testing.

### 2.4. Laboratory Analysis

Water samples were collected in 100 ml labeled sterile bottles and transported in cooler boxes with ice packs to the laboratory for processing within 6 hours of collection. Water samples were analyzed using the membrane filtration technique (EPA Method 1103.1) which is a quantitative method to quantify the actual number of fecal coliforms [29]. For chlorinated samples, sodium thiosulphate was added to the sample container to neutralize chlorine. Sterile distilled water (100 ml) was used as a negative control after every twentieth sample to ensure that the equipment had been adequately sanitized.

### 2.5. Membrane Filtration Technique (EPA Method 1103.1): Fecal Coliforms

Potatest field kit (Wagtech International, PTW10020) based on the field kit manual was used. The membrane filtration method was used to determine bacteriological water quality for fecal counts [17]. Potatest filtration sets were used to filter 100 ml of water sample through a 0.45 *μ*m pore size filter which retains bacteria that were present in the water sample, as also described by Köster et al. [30]. Samples were manually vacuum filtered. The filter was then transferred to a Petri dish containing absorbent pad and growth medium MLSB (Membrane Lauryl Sulphate Broth). MLSB contains lactose as the major carbon source, which during incubation is degraded to acid. Petri dish lid was then replaced and labeled with sample identification. Petri dishes were placed into the Petri dish rack ready to be incubated for 18 hours at different temperatures. An incubation temperature of 44°C was used for fecal coliforms. After incubation, yellow colonies grown on a plate were counted manually, and the concentration was reported as CFU/100 ml.

### 2.6. Salmonella

Water samples were processed using the method described in the EPA's Standard Analytical Protocol for *Salmonella typhi* in drinking water [31] to detect *Salmonella*. Potatest filtration sets were used to filter 100 mL of the water sample through a 0.45 *μ*m pore size filter which retains bacteria that were present in the water sample. Samples were manually vacuum filtered. The filter was transferred to a prepared nutrient agar (a wet bismuth-sulphite nutri disk) from the Potatest field kit. Nutri disk lids were then replaced and labeled with sample identification and then placed into the Petri dish rack ready to be incubated for 40–48 hours at 35°C incubation temperature. After incubation, *Salmonella* pathogens grew as black colonies with a surrounding metallic sheen resulting from hydrogen sulphide production and reduction of sulphite to black ferric sulphide. The concentration was reported as the colony-forming unit per 100 ml of water (CFU/100 ml).

### 2.7. Data Quality Management

Field data about practices around drinking water were collected by using a questionnaire adopting standard WASH knowledge, attitude, and practice questions [32]. Questionnaire data were entered using Excel Forms into Excel files. Laboratory data was entered into Microsoft Excel throughout the period of laboratory work. All data (field and laboratory) underwent multiple checking by all authors to check for completeness and any discrepancies. Upon cleaning, it was analyzed using R statistical software [33].

## 3. Results

### 3.1. Water-Handling Chains

**Figure 1:**
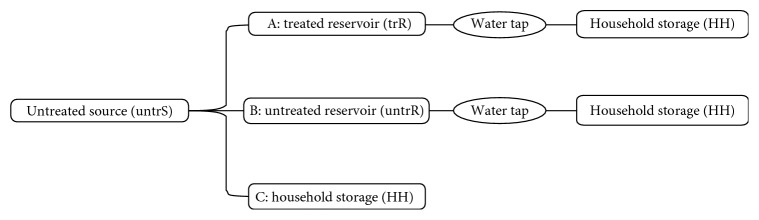
Three water-handling chains in Babati town: untreated-source-to-treated-reservoir-to-households (*untrS2trR2HH*); untreated-source-to-untreated-reservoir-to-households (*untrS2untrR2HH*); untreated-source-to-households (*untrS2HH*).

Three unique water-handling chains were discovered in this study. These chains were derived from analysis of water-handling practices that were reported by the studied households. From Figure 1, the chains are, namely, untreated source-untreated-reservoir-household, shortened as *untrS2untrR2HH*. The second chain is the untreated-source-treated-reservoir-household, shortened as *untrS2trR2HH*, and the third one was untreated-source-household, shortened as *untrS2HH*. Untreated sources include water sources such as dug wells, boreholes, and surface waters collected using gravity or electric pumps. In chain (A), (*untrS2trR2HH*), water flows through the pipeline to the reservoir for the treatment process to be taken and then distributed to the distribution points (taps) directly to be collected to the households for domestic uses and storage purposes for drinking. In chain (B) (*untrS2untrR2HH*), water is taken to reservoirs where it is not treated before distribution to the households. In chain (C) (*untrS2HH*), water from sources like surface water, dug wells, and boreholes is obtained by various ways including the dipping bucket to the wells or using pump (foot/hand pump) and then taken to the households (Figure 1).

### 3.2. Distribution of Chains

After specifying the water-handling chains, we analyzed the distribution of users of these chains. We found that 45.95% of households were using the *untrS2HH* chain, 27.03% of households were using the *untrS2trR2HH* chain, and 27.03% were using the *untrS2untrR2HH* chain. Numerically, most of the households were using the untreated-source-to-household (*untrS2HH*) chain, which is the most risky. Statistically, however, it was found that there was no evidence that the number of households using each chain differed (*x*^2^ = 5.2973, df = 2, *p*=0.07), meaning that households more or less involve themselves equally in employing the various water-handling chains.

### 3.3. Distribution of Types of Containers Used by Various Households

Households stored their water in three types of containers. There was a statistically significant difference of the number of households using various types of containers (*x*^2^ = 51.27, df = 2, *p* < 0.001), where 72% of the respondents stored their water in buckets, while 22% and 7% from the households stored their water predominantly in drums and pots, respectively. Households could use multiple storage containers.

### 3.4. Condition of Water-Handling Containers

When taking samples in household water-handling containers, their conditions were also observed. Generally, three types of containers are used as follows: 71.62% used buckets, 21.62% used drums/barrels, and 6.6% used traditional clay pots. These proportions were statistically significantly different using a chi-squared test (chi-squared = 69.276, df = 2, *p* < 0.001). The condition of containers observed in various households was whether the water containers were covered and clean, covered but dirty, and uncovered but clean. There was enough evidence to suggest that the number of households using each type of containers differed (*x*^2^ = 114.27, *p* < 0.001) where a high percentage (92%) of containers used by the households were found covered and clean, 5.4% were covered but dirty, and only 3% were found uncovered but clean. This reflects a high awareness among households in terms of ensuring that their water containers are kept clean and covered. The obvious fourth category of uncovered and dirty containers was not observed at all and hence the absence in our analysis.

### 3.5. Distribution of Water Treatment Means by Households

Water treatment is any process that removes contaminants and undesirable components from water, making it more acceptable for a specific end-use. Among the studied households, about 86% of them reported not treating their water, while only 14% boiled their water (*x*^2^ = 39.405, df *=* 1, *p* < 0.001). When water treatment behaviors were studied in the identified water-handling chains, it was found that most households using various chains did not treat their water before drinking (Table 1).

Respondents were also asked to give reasons for not treating the water, and most of them preferred not to treat water because it was expensive or because it rendered the water tasteless and/or treatment made water to have a smell (for chlorinated water). Chlorination practice was observed in the untreated-source-to-treated-reservoir-to-household chain (*untrS2trR2HH*), which is the evidence that chlorination was the method of choice in treating waters in reservoirs in Babati town. On the contrary, boiling of water was practiced by households getting their water from either the untreated-source-to-household chain (*untrS2HH*) or from the untreated-source-to-untreated reservoir-to-households (*untrS2untrR2HH*) chain.

### 3.6. Water-Handling Chains and Types of Containers

A breakdown of water-handling chains with regard to types of containers shows that the plastic bucket is the most used water container followed by water drums. Pots are only moderately used. The type of containers used per water-handling chain was statistically different for various water-handling chains (Pearson's chi-squared test: *x*^2^ = 12.017, df = 4, *p*=0.02) (Table 2). Most modern buckets and drums/barrels have lids, while clay pots almost always do not have lids. These structural features can have implications in the safety of the waters contained therein.

### 3.7. Microbiological Water Quality

Out of the 564 water samples collected, 485 (86%) samples were found contaminated and only 79 (14%) samples were free from all two microbial counts (i.e., fecal coliforms and *Salmonella*). Specifically, 412 (85%) of those contaminated were positive for fecal coliforms and 340 (70%) for *Salmonella typhi* count. The microbial counts were in colony-forming units per 100 ml. We further compared the amounts of these contaminations in the water-handling chains to see the level of risk among the chains.  Table 3 is an ANOVA table which shows that there was a statistically significant difference in coliforms among the chains (*p* value = 0.01). Tukey's post hoc test to test differences among chains is shown in Table 4 and shows that there were significantly higher fecal coliforms counts within the chains of the untreated-source-to-households (*untrS2HH*) compared to the other chains (*p*=0.02) as reflected by the large negative size of the difference.

**Figure 2:**
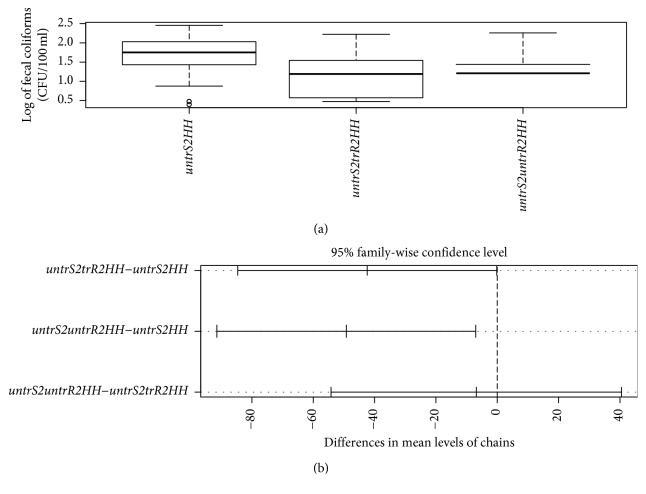
Boxplot (a) and pairwise comparison (b) of fecal coliforms counts (CFU/100 mls) in various water-handling chains (*untrS2HH* = untreated source to households; *untrS2trR2HH* = untreated source to treated reservoir to households; *untrS2untrR2HH* = untreated source to untreated reservoir to households).

The water-handling chain from the untreated-source-to-treated-reservoir-to-households (*untrS2trR2HH*) had the lowest median levels of fecal coliforms of the three chains (Figure 2 and Table 4) though the difference between this chain and the untreated source to untreated reservoir to households was not statistically significant (*p*=0.94).

Comparison of fecal coliforms among water-handling chains revealed that the largest difference was between the untreated-source-to-untreated-reservoir-to-household (*untrS2untrR2HH*) chain and the untreated-source-to-household (*untrS2HH*) chain, followed by the untreated-source-to-treated-reservoir-to-household (*untrS2trR2HH*) and the untreated-sources-to-household (*untrS2HH*) chain. The smallest difference is between the *untrS2untrR2HH* and the *untrS2trR2HH* chains providing evidence that the treatment of water that is usually done at the reservoir and household behaviors around water treatment are effective in reducing the fecal coliforms (Table 4).

**Figure 3:**
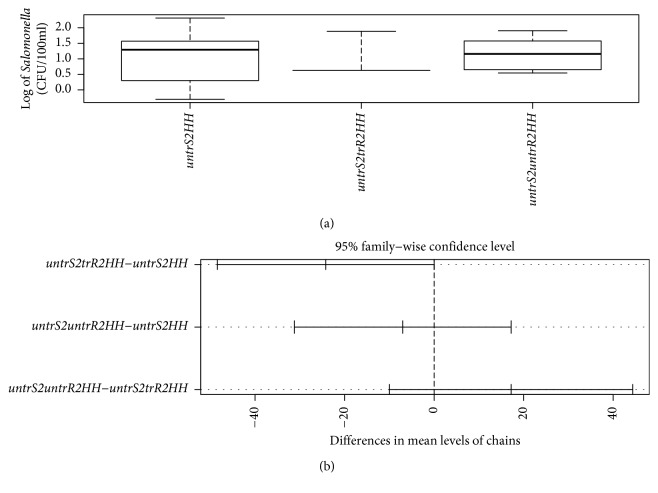
Boxplot (a) and pairwise comparison (b) of *Salmonella typhi* counts (CFU/100 mls) in water-handling chains (*untrS2HH* = untreated source to households; *untrS2trR2HH* = untreated source to treated reservoir to households; *untrS2untrR2HH* = untreated source to untreated reservoir to households).

In terms of *Salmonella typhi*, Table 5 is an ANOVA table showing that there was no statistically significant difference among the chains in terms of the *Salmonella* counts (*p*=0.06) (also reinforced in Figure 3). The chain with the untreated source to households (*untrS2HH*) had the highest counts compared to the other water-handling chains. The chain with the lowest count was the one with the untreated source to treated reservoir to households, again pointing to the evidence of effectiveness of water treatment.

Table 6 is Tukey's post hoc test to test the chain-to-chain differences. A comparison of levels of *Salmonella* for each chain revealed that the *untrS2trR2HH*-*untrS2HH* pair and the *untrS2untrR2HH*-*untrS2trR2HH* pair had the largest difference (Table 6 and Figure 3) with the lowest difference being between the *untrS2untrR2HH*-*untrS2HH* chains. People involved in the *untrS2HH* chain are having the most exposure to *Salmonella typhi*.

### 3.8. Role of Water-Handling Chains and Type of Containers in the Contamination Profile

**Figure 4:**
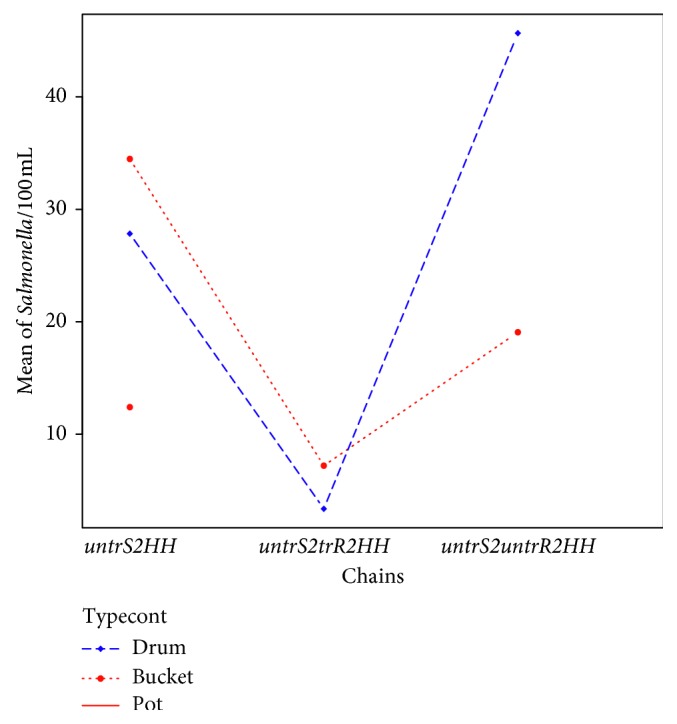
Interaction between container type, water-handling chains, and mean of *Salmonella* contamination in CFU/100 mls (*untrS2HH* *=* untreated source to households; *untrS2trR2HH* *=* untreated source to treated reservoir to households; *untrS2untrR2HH* *=* untreated source to untreated reservoir to households; *typecont* = type of container).

The chain with the lowest count of the microbial profile was the untreated source to treated reservoir to the household (*untrS2trR2HH*) pointing to the possible effectiveness of the routine water treatment conducted by the Babati Water and Sanitation Authority (BAWASA).  Figure 4 shows the relationships between the *Salmonella* contamination, water containers, and the water-handling chains. The chain *untrS2trR2HH* is therefore a baseline (since it has the lowest contamination), and the roles of container types and container conditions with respect to the two other chains (*untrS2HH* and *untrS2untrR2HH*) are shown. We see that, within the *untrS2untrR2HH* chain, the drum had the most concentrations of *Salmonella typhi*, whereas, within the *untrS2HH* chain, the culprits were buckets and drums (Figure 4).

**Figure 5:**
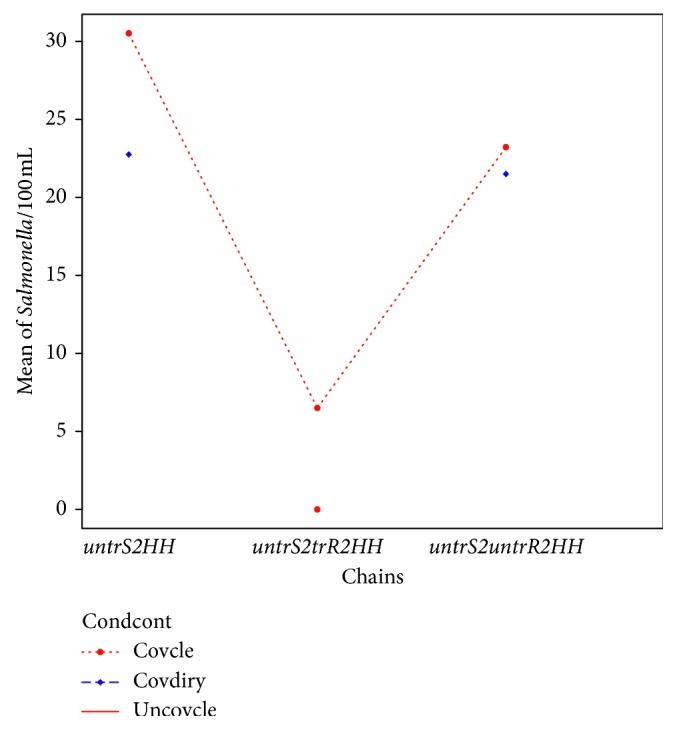
Interaction between container condition, water-handling chains, and *Salmonella* counts CFU/100 mls (*untrS2HH* = untreated source to households; *untrS2trR2HH* = untreated source to treated reservoir to households; *untrS2untrR2HH* = untreated source to untreated reservoir to households; *covcle* = covered and clean; *covdir* = covered and dirty; *uncovcle* = uncovered but clean).

In terms of the condition of containers, as can be seen from Figure 5, containers that were covered and seemingly clean also had a substantial count of *Salmonella* in this chain, and this is an interesting observation since we would expect what is “clean and covered” to have the lowest amount of contamination. Surprisingly, containers that were covered and seemingly dirty had lower amount of contamination despite that the numerical difference was not statistically significantly different between the two conditions of the containers (*p*=0.88). Moreover, within the *untrS2HH* chain, the covered and clean container actually had the highest counts of *Salmonella* in comparison to the covered and dirty containers (Figure 5).

These seemingly contradictory findings underscore our hypothesis that the safety of water is a function of the entire set of activities happening in the entire water-handling value chain. Contamination can happen at many points within the chain making it possible that even covered and clean containers can have higher contaminations than uncovered and dirty containers. In fact, if the contamination happens along the chain, it is possible that the coverage that happens in the household vessels could even promote the multiplication of microbes because of favorable conditions such as temperatures.

## 4. Discussion

The present study aimed at characterizing unique water-handling chains and assessing their water quality in Babati town in Tanzania. Three water-handling chains which included the untreated source to treated reservoir to household (*untrS2trR2HH*), untreated source to untreated reservoir to household (*untrS2untrR2HH*), and untreated source to household (*untrS2HH*) were characterized. It was found that most people taking water from untreated water source straight to households (chain *untrS2HH*) were more vulnerable to infectious disease compared to the other chains because the water they consumed was never treated and thus was safe to human consumption as reported in other studies [34–36]. Sourcing water from untreated sources could potentially put consumers at risk of typhoid fever due to direct access of people and animals to water sources [37, 38].

The study also revealed that buckets and drums which were covered and clean were mostly used. Findings at the households revealed that few people were using the uncovered container implying some adherence to hygienic behaviors. The way communities use water-handling containers that have important implications to hygiene, as reported by Sobsey et al. [10], Singh et al. [39], Pickering et al. [40], and Devamani et al. [41] who reported poor hygiene as an important factor for disease spread within the communities where water quality was affected by dipping hands into water when fetching.

In this study, most households did not treat the water and stored it mainly in buckets and drums. This situation of storing untreated water could pose a high risk of contamination due to high concentration of microbes in this stationary water, a situation also reported by Wright et al. [42], Gundry et al. [43], and Sharma et al. [44]. These commentators reported that contamination was mostly found in storage containers compared to the distribution points and reservoirs mainly because of fresh bacterial contamination of the storage vessels. Indeed, there was evidence from Lesotho, Nigeria, and New Zealand for the fecal contamination in domestic storage vessels being of human origin, while that in public water supply is more likely to be predominantly of animal origin [45–48]. For example, a study in Zambia concludes that boiling process alone could not reduce microbial contamination because recontamination could also occur due to dipping of contaminated vessels into drinking water when fetching it [49].

The low numbers of households treating their water in Babati are striking, and qualitative methods would probably give more definitive answers as to why many people do not treat their water. In the literature, there are several reasons for people not treating their water ranging from being tasteless to being smelly and at times just costly [17, 50, 51].

Our study has also revealed that contaminations by fecal counts and *S. typhi* were all above the Tanzania and WHO standard value of 0 CFU/100 mls, and this highlights that most people are consuming unsafe water. This calls for precautionary measures to be taken during the on-site risk assessment from collection to point of use to prevent cases of waterborne diseases. Our characterization of the unique water-handling chain is a novel result which can clearly help guide policies and plans in curbing the threats of water-borne diseases. With some investment, households using various chains could be mapped and intervened to rid them of the risks associated with consuming unsafe water. Babati town is finalizing its urbanization plan to a new site, and information from this study is already helping in the design of this future city.

Future characterization of water-handling chains using longitudinal studies and applying other visual tools such as spatial visualization could greatly enhance our understanding of water-handling chains with respect to seasons and geographical nature.

## 5. Conclusion

In this study, we found that three patterns of water handling were commonly practiced in the studied area with a revelation of poor water storage and handling as evidenced by the microbial profiles performing poorly against national and WHO standards. The water-handling patterns did harbor active population of microorganisms that could threaten the public health. Even though treated water may be free of fecal indicator organisms, water-handling practices done from source, collection, and transportation to the households may hinder water quality and hence increase vulnerability to waterborne diseases. Knowledge on proper protection of the source, reservoir, and distribution points and regularly monitoring throughout the distribution chain and encouraging the community to use home-based water treatment system like boiling and safe storage and fetching practices could reduce diarrheal and other waterborne diseases in communities and households of growing cities in developing countries. Results from our study inform urban and city authorities as well as a water and sanitation authorities which are the main bodies responsible for maintaining drinking water quality. It also informs individual consumers about the dangers of using a particular chain, and therefore, this study is ideal for general consumption as well. WASH lesson plans could be updated to include this knowledge, which the study has added. At the top level, this new knowledge can better guide public health and public health policy interventions to reduce the health impact of waterborne diseases.

## Figures and Tables

**Table 1:** Water treatment behavior among users of various water-handling chains.

Water-handling chains	Water treatment	
	Boiling (%)	No treatment (%)
*untrS2HH* (untreated source to household)	10.8	35.1
*untrS2trR2HH* (untreated source to treated reservoir to household)	0.0	27.0
*untrS2untrR2HH* (untreated source to untreated reservoir to household)	2.7	24.3
*p* value (chi-squared = 8.453, df *=* 2) = 0.0146		

**Table 2:** Container types among users of various water-handling chains.

Water-handling chains	Container type		
	Bucket (%)	Drum (%)	Pot (%)
*untrS2HH* (untreated source to household)	31.1	8.1	6.8
*untrS2trR2HH* (untreated source to treated reservoir to household)	17.6	9.5	0.0
*untrS2untrR2HH* (untreated source to untreated reservoir to household)	23	4.1	0.00
*p* value (chi-squared = 12.017, df = 4) = 0.01722			

**Table 3:** Fecal coliforms among various chains.

	df	Sum of squares	Mean squares	*F* value	*p* (>*F*)
Chains	2	39028	19514	4.983	0.00944
Residuals	71	278049	3917		

**Table 4:** Fecal coliforms among paired chains.

	Difference	Lower limit	Upper limit	Adjusted *p* values
*untrS2trR2HH-untrS2HH*	−42.39412	−84.60919	−0.1790449	0.0488051
*untrS2untrR2HH-untrS2HH*	−49.21912	−91.43419	−7.0040449	0.0182864
*untrS2untrR2HH-untrS2trR2HH*	−6.82500	−54.19737	40.5473706	0.9365991

**Table 5:** *Salmonella* counts among various chains.

	df	Sum of squares	Mean squares	*F* value	*p*(>*F*)
Chains	2	7436	3718	2.886	0.0624
Residuals	71	91470	1288		

**Table 6:** *Salmonella typhi* counts among paired chains.

	Difference	Lower limit	Upper limit	Adjusted *p* value
*untrS2trR2HH-untrS2HH*	−24.208824	−48.421747	0.00410	0.0500482
*untrS2untrR2HH-untrS2HH*	−7.008824	−31.221747	17.20410	0.7683792
*untrS2untrR2HH-untrS2trR2HH*	17.200000	−9.970948	44.37095	0.2898869

## Data Availability

The datasets used and/or analyzed during the current study are available from the corresponding author on reasonable request.

## List of abbreviations

CFU: Colony-forming unit
Covcle: Covered and clean
Covdiry: Covered but dirty
HH: Household
LiSBE: School of Life Sciences and Bioengineering of NM-AIST
MEWES: School of Materials, Energy and Water Engineering and Sciences of NM-AIST
MLSB: Membrane Lauryl Sulphate Broth
NM-AIST: Nelson Mandela African Institution of Science and Technology
Notr: No treatment
trR: Treated reservoir
Uncovcle: Uncovered but clean
untrR: Untreated reservoir
untrS: Untreated source
*untrS2HH*: Untreated source to household
*untrS2trR2HH*: Untreated source to treated reservoir to household
untrS2untrR2HH: Untreated source to untreated reservoir to household.
